# Effect of a Nursing intervention on the uncertainty of family members in Intensive Care

**DOI:** 10.15649/cuidarte.3220

**Published:** 2024-05-27

**Authors:** Diego Omar Pérez Campos, Alejandra Fuentes-Ramírez

**Affiliations:** 1 Universidad de La Sabana, Chía, Colombia. omarperezcam@gmail.com Universidad de la Sabana Universidad de La Sabana Chía Colombia omarperezcam@gmail.com; 2 Universidad de La Sabana, Chía, Colombia. alejandra.fuentes@unisabana.edu.co Universidad de la Sabana Universidad de La Sabana Chía Colombia alejandra.fuentes@unisabana.edu.co

**Keywords:** Critical Care, Nursing Care, Uncertainty, Family, Life-Changing Events, Cuidados Críticos, Atención de Enfermería, Incertidumbre, Familia, Acontecimientos que Cambian la Vida, Cuidados Críticos, Cuidados de Enfermagem, Incerteza, Família, Acontecimentos que Mudam a Vida

## Abstract

**Introduction:**

Family members of patients admitted to an Intensive Care Unit present high uncertainty level due to not knowing what is happening and to not having clear details about the related events; therefore, interventions are required to allow modulating those levels.

**Objective:**

To evaluate the effect of an educational Nursing intervention compared to conventional care on the uncertainty of family members of patients hospitalized in an ICU.

**Materials and methods:**

An experimental study with a sample comprised by 132 relatives of patients admitted to an ICU, randomly distributed in four Solomon groups (33 in each group). The Nursing intervention based on the concepts of the Uncertainty in Illness Theory was applied to both experimental groups and devised under the Whittemore and Grey parameters with three moments: assessment; education about the relative's hospitalization in the ICU; and accompaniment. This was done with pre-assessments for two groups and post-assessments for the four groups, using the PPUS-FM Uncertainty Scale. The data were analyzed by means of descriptive statistics and respective non-parametric analyses. The study took into account the ethical principles in research.

**Results:**

The family members in the experimental groups presented a lower final uncertainty level when compared to the control groups, with a difference of 73.04 points and a p-value of 0.001.

**Discussion:**

Standardized interventions and under a theoretical model allow reducing uncertainty in relatives of patients in ICUs.

**Conclusions:**

The Nursing intervention based on the Uncertainty theory allows reducing uncertainty in relatives of patients hospitalized in an Intensive Care Unit.

## Resumen

**Introducción:**

Los familiares de pacientes que ingresan a una UCI presentan altos niveles de incertidumbre, dados por el desconocimiento de lo que está sucediendo y por no tener detalles claros sobre los eventos relacionados; por lo tanto, es necesario realizar intervenciones que permitan modularla.

**Objetivo:**

Evaluar el efecto de una intervención educativa de enfermería comparada con los cuidados convencionales sobre la incertidumbre de los familiares de pacientes hospitalizados en una UCI.

**Materiales y métodos:**

Estudio experimental con una muestra de 132 familiares de pacientes ingresados a una UCI, distribuidos aleatoriamente entre cuatro grupos de Solomón (33 en cada grupo), A los dos grupos experimentales se les aplicó la intervención basada en los conceptos de la teoría de Incertidumbre sobre la Enfermedad y se construyó bajo los parámetros de Whittemore y Grey con tres momentos: valoración, educación sobre la estancia del familiar en la UCI y acompañamiento. Lo anterior se implementó con evaluaciones previas para dos grupos y posteriores para los cuatro grupos de la Escala de Incertidumbre PPUS-FM. Los datos se analizaron por medio de estadística descriptiva y análisis no paramétricos respectivos. El estudio tuvo en cuenta los principios éticos en investigación.

**Resultados:**

Los familiares de los grupos experimentales presentaron un nivel de incertidumbre final menor en comparación con los grupos control, con una diferencia de 73.04 puntos y valor p de 0,001.

**Discusión:**

Las intervenciones estandarizadas y bajo un modelo teórico permiten reducir la incertidumbre de familiares de pacientes en una UCI.

**Conclusiones:**

La intervención de enfermería basada en la teoría de la Incertidumbre permite disminuir la incertidumbre en los familiares de pacientes hospitalizados en una UCI.

## Resumo

**Introdução:**

Familiares de pacientes internados em UTI apresentam altos níveis de incerteza, dado o desconhecimento do que está acontecendo e não tendo clareza sobre os eventos relacionados; portanto, é necessária a realização de intervenções que permitam sua modulação.

**Objetivo:**

Avaliar o efeito de uma intervenção educativa de enfermagem comparada aos cuidados convencionais na incerteza de familiares de pacientes internados em UTI.

**Materiais e métodos:**

Estudo experimental, com amostra de 132 familiares de pacientes internados em UTI, distribuídos aleatoriamente em quatro grupos Solomon (33 para cada grupo), sendo a intervenção baseada nos conceitos da teoria aplicada aos dois grupos experimentais. de incerteza da doença e foi construído sob os parâmetros de Whittemore e Gray com três momentos: avaliação, educação sobre a permanência do familiar na UTI e apoio. Com avaliações antes para dois grupos e depois para os quatro grupos da Escala de Incerteza -PPUS-FM. Os dados foram analisados com estatística descritiva e respectivas análises não paramétricas. O estudo levou em consideração os princípios éticos em pesquisa.

**Resultados:**

Os familiares dos grupos experimentais apresentaram menor nível de incerteza final em comparação ao grupo controle, com diferença de 73,04 pontos com valor de p de 0,001.

**Discussão:**

Intervenções padronizadas sob modelo teórico permitem reduzir a incerteza dos familiares de pacientes internados em UTI.

**Conclusões:**

A intervenção de enfermagem baseada na teoria da incerteza permite reduzir a incerteza nos familiares de pacientes internados em UTI.

## Introduction

Intensive Care Units (ICUs) are spaces created to care for patients with and acute chronic diseases that require specialized assistance; therefore, ICUs are usually closed units where family members are separated from the patient at admission and are moved to the background, in order to focus the therapeutic efforts on the patient. However, hospitalization in an Intensive Care Unit not only affects the patient but also their family members, who present needs that should be met^1^.

If not solved, the family members' needs increase the uncertainty level, which causes in them countless problems, both ofa physical and of mental and social natures. It is important to note that the family is an integral component of Nursing assistance as a care subject, reason why the professionals are required to apply interventions that allow modulating the uncertainty generated in the relatives, in addition to being a constitutional right^2^^,^^4^.

It is also relevant to state that the conventional care offered to family members by Nursing professionals is solely focused on making them sign the different consent forms at admission and, subsequently, on communicating with them to report if the patient has any type of diagnostic test scheduled that requires their presence.

The uncertainty generated in the family members at ICU admission is documented^5^^,^^6^ and middle-range theories such as Merle Mishel's instruct the professionals based on disciplinary knowledge about the measures to be adopted to care for the family member during this period^3^^,^^7^; it is because of the theory that the source of the problem can be understood and, in the same way, how to modulate it.

Given the aforementioned, the objective of the current study was to assess the effect of an educational Nursing intervention compared to conventional care on uncertainty in family members of patients hospitalized in an ICU.

### Uncertainty of family members in an ICU

Uncertainty has its onset when the loved one is admitted to the ICU; due to the number of unexpected events, family members become incapable of attributing clear meanings to the situation^3^. Likewise, among the concepts proposed by Mishel in her theory, the structure sources represent the existing resources to assist the person in interpreting what is happening^8^, which is comprised by:

- Credible authority: it is the degree of trust deposited by the person on the health personnel caring for them, which are Nursing professionals in this case, for being the health staff sharing the most time with the patient when compared to the other professionals^9^^,^^10^.
- Social support: the social support received exerts an influence on the uncertainty levels when it helps people interpret the meaning of the facts. Nursing professionals understand the concept of uncertainty and the effects it causes on family members; in addition, they apply suitable interventions so that the relatives can modulate it.
- Education: it reduces the uncertainty because, by having a knowledge base, it is possible to attribute meaning to the fact and to better understand it. It is characterized as with the objective of providing tools to the family members so that they make decisions based on the information offered about the treatment and the subsequent interventions and, thus, being able to include education about any aspects unknown by the family members about the ICU operation, components and health/support personnel, in addition to the care plan for the loved one. It is a prerequisite to elaborate clear meanings about the current event.

**Figure 1:**
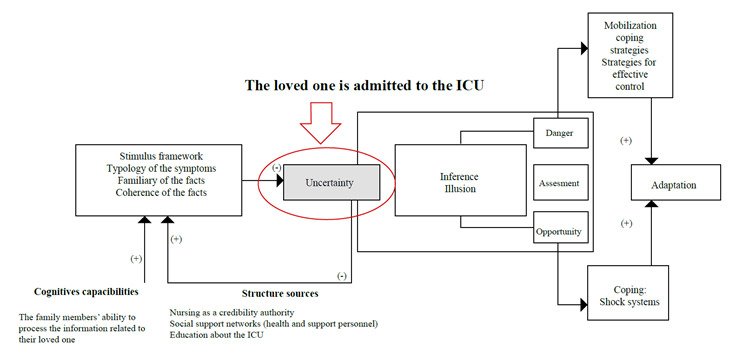
Application of the concepts of uncertainty in family members of patients hospitalized in an ICU

Source: Adapted from Merle Mishel's Uncertainty in Illness Theory.

In relation to the family members' cognitive capabilities, they can vary according to each person; uncertainty has its onset when the loved one is admitted to the ICU; due to the number of unexpected events, family members become incapable of attributing clear meanings to the situation^3^ (see Figure 1).

## Materials and methods

Design: Experimental study with four Solomon groups: two experimental and two control, with random allocation.

Participants: The population consisted of the adult family members of patients hospitalized for the first time in the ICU for adults of a health institution from the city of Neiva, Colombia, during the 2020-2021 period.

Inclusion criteria: Adult family members of patients hospitalized for the first time in an ICU.

Exclusion criteria: Adult family members of ICU-admitted patients that were not located or were not in due physical and mental conditions to answer during data collection.

Sample size: The sample was comprised by 132 family members; each group consisted of 33 relatives^11^. Allocation to the groups was defined in a simple random way, taking the following scheme into account (see Table 1).

**Table 1:** Solomon group experimental design scheme

#	Group	Baseline assessment	Intervention	Final assessment
1	Experimental 1	x	x	x
2	Experimental 2		x	x
3	Control 1	x		x
4	Control 2			x

Measurement variables: Disease uncertainty was assessed with Merle Mishel's Uncertainty in Illness scale, family member form (PPUS-FM), which has 31 items with a Cronbach's alpha of 0.81. This scale classifies the uncertainty level according to its final score, as follows: low UL with <61 points, fair UL with 61-89 points; and high UL with >89 points^9^. In addition, a sociodemographic data form was also included.

Randomization: The group allocation for the family members that met the inclusion criteria and gave the consent to participate in signing was performed in a simple random way, with a list of random numbers in Excel.

Procedure: The control groups were offered the conventional intervention, which consists in the interventions performed by the Nursing personnel to serve the family members of the individuals hospitalized in the ICU for adults. These care measures are focused on making the family member sign informed consent forms at the patient's admission and, subsequently, communicating with them to report if the patient has some type of diagnostic test scheduled that requires their presence.

**Figure 2:**
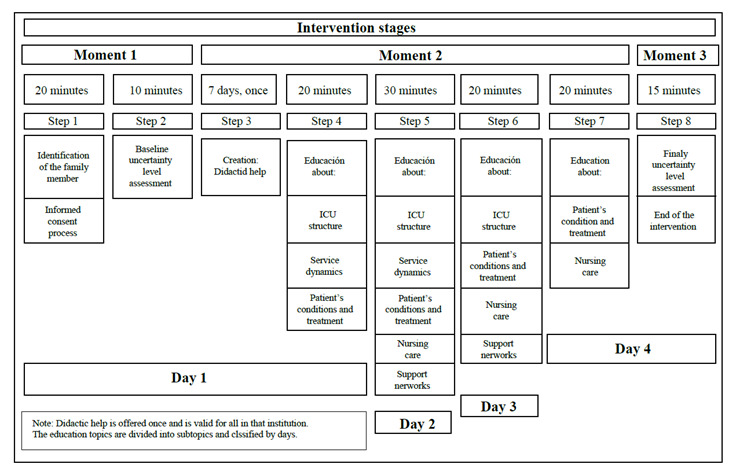
Intervention stages

The Intervention Group was offered the educational Nursing intervention called “Acompañándote en la UCI” (“Being with you in the ICU”), which was prepared taking into account the concepts of cognitive capabilities and structure sources from Merle Mishel's Uncertainty in Illness Theory and Robin Whittemore's guidelines for the systematic development of Nursing interventions^12^. The Nursing intervention for relatives of patients hospitalized in the ICU is applied for four days and instructs on the following: ICU structure; service dynamics; patient's conditions and treatment; support networks; and Nursing care Figure 2. To ensure reliability of the intervention, an operations manual was prepared that explains in detail each moment and contains a checklist to ensure that the intervention is applied.

Data analysis: During the data analysis, an Excel document was generated in Google Forms, which was subjected to verification and debugging of incorrect and missing data; subsequently, the Statistical Package for Social Science, SPSS® (version 26) was used, under license by *Universidad de La Sabana*. The database was stored in Mendeley Data17^13^. Normality was verified with the Shapiro-Wilk test. The result indicated that the measurements in the groups do not correspond to normal distribution. The analysis of the data obtained was performed through two approaches:

1. An intra-group approach that evaluated changes in the uncertainty levels.
2. An inter-group approach that compared changes in the uncertainty levels across the groups.

The analysis of the results in the intra-group analysis resorted to the Wilcoxon test for the experimental and control groups. In addition, the Mann-Whitney U test was performed in the analysis of the inter group results and, finally, the ANOVA test was applied in Experimental Group 1 and Control Group 1, in order to verify the need to collect a baseline measure or not.

### Ethical considerations

The research observed the ethical principles ofresearch as per Resolution No. 8430 of 199314, as minimal risk. The international and national guidelines for research in health were respected. It was approved by the Research and Ethics Subcommission of Universidad de La Sabana via Minute No. 011 of 2021; it was also approved by the institution where the sample was collected. All the participants agreed to be included in the study and signed the informed consent form. Environmental care measures were taken into account.

## Results

The study population consisted of four groups, each one with 33 family members of patients hospitalized in an ICU from the city, therefore accounting for a total of 132 participants. The sociodemographic data can be consulted in Table 2.

**Table 2:** Sociodemographic characterization by groups

	Experimental Group 1 33	Experimental Group 2 33	Control Group 1 33	Control Group 2 33	p-value
Age					0.95
15-44	66.66 (22)	60.60 (20)	57.57 (19)	63.63 (21)	
45-64	27.27 (9)	30.30 (10)	33.33 (11)	30.30 (10)	
>65	6.06 (2)	9.09 (3)	9.09 (3)	6.06 (2)	
Kinship with the patient					0.13
Father/Mother	6.06% (2)	9.09% (3)	12.12% (4)	12.12% (4)	
Brother/Sister	6.06% (2)	9.09% (3)	6.06% (2)	18.18% (6)	
Spouse	9.09% (3)	24.24% (8)	24.24% (8)	6.06% (2)	
Son/Daughter	60.61% (20)	48.48% (16)	39.39% (13)	39.39% (13)	
Other	18.18% (6)	9.09% (3)	18.18% (6)	24.24% (8)	
Gender					0.06
Male	9.09% (3)	36.36% (12)	30.30% (10)	24.24% (8)	
Female	90.91% (30)	63.63% (21)	69.69% (23)	75.75% (25)	
Strata					0.52
Stratum 1	45.45% (15)	42.42% (14)	51.51% (17)	63.63% (21)	
Stratum 2	42.42% (14)	48.48% (16)	45.45% (15)	27.27% (9)	
Stratum 3	9.09% (3)	9.09% (3)	3.03% (1)	9.09% (3)	
Stratum 4	3.03% (1)	0% (0)	0% (0)	0% (0)	
Occupation					0.42
Employee	12.12% (4)	18.18% (6)	9.09% (3)	6.06% (2)	
Unemployed	0% (0)	3.03% (1)	0% (0)	3.03% (1)	
Freelance worker	15.15% (5)	45.45% (15)	36.36% (12)	39.39% (13)	
House chores	63.63% (21)	27.27% (9)	51.51% (17)	42.42% (14)	
Retiree	3.03% (1)	3.03% (1)	3.03% (1)	3.03% (1)	
Other	6.06% (2)	3.03% (1)	0% (0)	6.06% (2)	
Schooling					0.71
Can read/write	9.09% (3)	3.03% (1)	3.03% (1)	9.09% (3)	
Elementary School	27.27% (9)	27.27% (9)	45.45% (15)	36.36% (12)	
High School	42.42% (14)	51.51% (17)	39.39% (13)	48.48% (16)	
Technician/	15.15% (5)	12.12% (4)	12.12% (4)	6.06% (2)	
Technologist					
Higher Education	6.06% (2)	6.06% (2)	0% (0)	0% (0)	
Graduate Studies	0% (0)	0% (0)	0% (0)	0% (0)	
Religion					0.85
Catholics	93.93% (31)	96.96% (32)	93.93% (31)	93.93% (31)	
Protestants	3.03% (1)	3.03% (1)	3.03% (1)	6.06% (2)	
Evangelical	3.03% (1)	0% (0)	3.03% (1)	0% (0)	
Other	0% (0)	0% (0)	0% (0)	0% (0)	
Marital Status					0.74
Single	24.24% (8)	18.18% (6)	9.09% (3)		
Married	21.21% (7)	18.18% (6)	36.36% (12)		
Consensual union	42.42% (14)	54.54% (18)	51.51% (17)		
Separated/Divorced	3.03% (1)	3.03% (1)	0% (0)		
Widowed	9.09% (3)	6.06% (2)	3.03% (1)		
Baseline Uncertainty					0.14**
Mean ± SD	125 ±15	Not measured	132 ±14		
Minimum - Maximum	77 - 154	Not measured	103 - 154		
Mediana	125	Not measured	128		
Final Uncertainty					0.00**
Mean ± SD	59 ±17	55 ±14	126 ±22		
Minimum - Maximum	31 - 108	31 - 93	63 - 155		
Mediana	62	59	121		

*Fisher's Exact Test p<0.05: significant difference. **ANCOVA p<0.05: significant difference.

The participants' mean age was 41 years old, varying from 18 to 85; more than 63.62% in each group were women; 47% were the patients' children; and, regarding religion, Catholicism predominated with more than 93.92% in each group. More than 87.86% of the participant belonged to Stratus 2 or lower; with house chores ranking first in each group, followed by freelance workers, with the exception of the post-intervention Experimental group, where freelance workers prevailed, followed by house chores. In terms of schooling, more than 69.68% of the participants from each group have from Elementary to High School level. Regarding the participants' marital status, more than 42.41% in all groups are in consensual unions, followed by married individuals, as can be seen in Table 2. According to the aforementioned, no major difference between the groups is noticed.

The data obtained by the groups that were offered the Nursing intervention and conventional care are presented below (see Table 2).

Based on Table 2, it is noticed that the experimental groups presented high uncertainty with a mean Uncertainty Level (UL) = 125 points in the baseline assessments; in turn, they obtained low uncertainty with a mean UL <60 points. There is no larger difference in final uncertainty between the experimental groups, which might indicate that previous exposure (baseline assessment) to the form does not affect the result.

The following is observed in Table 2 regarding the control groups: they presented high uncertainty with a mean UL <125 points in the baseline and final assessments, respectively. In addition, they present very similar distributions, which might indicate that this does not affect the previous exposure result (baseline assessment) of the form.

The data obtained in the comparison between the effect of a Nursing intervention and conventional care on the uncertainty level in family members of people hospitalized in an ICU are presented below.

La incertidumbre se clasifica según su puntaje final en bajo NI = < 61 puntos, regular NI = 61-89 puntos Uncertainty is classified according to its final score, as follows: low UL with <61 points, fair UL with 61- 89 points; and high UL with >89 points 14. The uncertainty classification across the experimental and control groups is presented below, according to the aforementioned categorization (see Table 3).

**Table 3:** Baseline and final uncertainty classification across the experimental and control groups

Uncertainty Level	Baseline uncertainty classification		Final uncertainty classification	
	Experimental Group	Control Group	Experimental Group	Control Group
Low	0% (0)	0% (0)	46.97% (31)	0% (0)
Fair	3.03% (1)	0% (0)	48.48% (32)	4.55% (3)
High	96.97% (32)	100% (33)	4.55% (3)	95.45 (63)

*Wilcoxon p: 0.001 Experimental Group. ** Wilcoxon p:0.100 Control Group.

**Figure 3:**
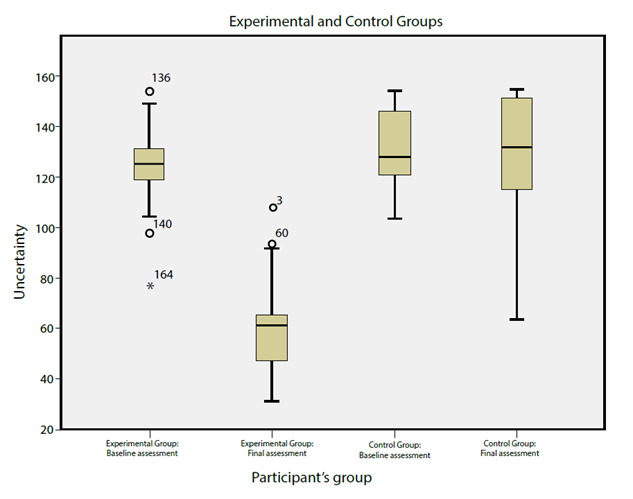
Box diagram: Baseline and final uncertainty across the groups

Table 3 shows that baseline uncertainty is almost as high in the experimental group (96.97%) when compared to the control group. In turn, final uncertainty in the Experimental Group differs by 95.45% when compared to the Control Group; in this case, the Experimental Group reaches from fair to low uncertainty and the Control Group continues with uncertainty. Finally, it can be inferred that nearly 50% of the participants from the experimental group went from high to low uncertainty and that the rest dropped from high to fair uncertainty (see Figure 3).

*P<0.05: significant difference, ANCOVA.

Figure 3 shows that the Experimental Group presented a reduction of nearly 50% in final uncertainty when compared to the baseline value; in turn, final uncertainty slightly increased its mean value in the control groups, which allows inferring that the Nursing intervention reduces approximately 50% the uncertainty levels when compared to conventional care. In addition, receiving conventional care can generate an increase in the uncertainty level.

The central tendency measures observed for the experimental and control groups by each of the 4 subgroups are as follows: Experimental Group 1 starts with a mean uncertainty of 125 and ends with 59 (with a 66-point difference), whereas Control Group 1 has a baseline mean of 132 and ends with 126 (with a 6-point difference). There is a reduction in both groups; however, the one found in Experimental Group 1 exceeded 50%, whereas it was quite minimal in Control Group 1. In addition, it is noticed that Experimental Group 1 ends with a mean uncertainty of 59, whereas Experimental Group 2 ends with a mean of 55, and standard deviations of 17 and 14, respectively. It is also noticed that Control Group 1 ends with a mean uncertainty level of 126, whereas Control Group 2 ends with a mean of 136 and standard deviations of 22 and 20, respectively. This allows us to infer that the experimental and control groups present similar results; therefore, it would be necessary to resort to Solomon groups because there was no variation with prior knowledge about the uncertainty form.

The Shapiro-Wilk test indicates non-normal distributions; therefore, the hypothesis test is performed with Wilcoxon's, noticing that a statistically significant difference between baseline and final uncertainty is evidenced in Experimental Group 1 (p=0.001), which indicates that the intervention was effective; in turn, no significant differences are observed between baseline and final uncertainty in Control Group 1 (p=0.100).

Taking into account Mann-Whitney's U test, the distribution corresponding to final uncertainty is not statistically different either between the experimental groups (p=0.314) or in the control groups (p=0.078). Therefore, it is not necessary to resort to Solomon groups, and the results can only be differentiated with the final assessment.

Finally, the ANCOVA test was applied considering final uncertainty as dependent variable, Experimental Group 1 and Control Group 1 as independent variables and baseline uncertainty as their covariate; reasserting that there is a statistical difference (p=0.001) in the final uncertainty level among the experimental and control groups; in addition, it is evidenced that it is not necessary to consider any baseline assessment because there is no statistical difference: p=0.143.

## Discussion

The study shows the effectiveness of the Nursing intervention for family members of patients hospitalized in an ICU on uncertainty, grounded on Merle Mishel's middle-range theory^13^. This research allows confirming the concepts of the theory and contributes more scientific knowledge about the phenomenon; in addition, the uncertainty level is significantly reduced in this study when compared to the family members that were offered conventional care.

Family members have their own care needs, which should be included in Nursing assistance as an integral care component^4^^,^^14^^,^^15^. The caregivers were mostly women, as can also be observed in different studies^3^^,^^6^^,^^16^^-^^18^. However, we also notice men emerging as caregivers, which slights reasserts the social change in terms of responsibility.

Given the above, it can be noticed that when their loved one is hospitalized, family members star to present different needs, especially regarding information, which is focused on their lack of knowledge about ICUs, their relative's health status, treatment and care they would be offered and whether they will survive, as is the case in the study by Taboada^6^. The aforementioned is worsened when family members lack any credible authority providing them proper education, taking into account how information is conveyed and the words used, among other details^4^.

By applying Merle Mishel's middle-range theory and its assessment scale for family members it was possible to easily evidence the phenomenon. The average-high baseline uncertainty levels are in line with other studies such as the one by Taboada and Inna^6^^,^^7^; in turn, they presented an average uncertainty level in the study by Massa^3^. However, this is attributed to the fact that it is a new event for the family members, reason why they lack proper tools to assign a clear meaning to the event^6^^,^^13^.

The results show that conventional care is not effective, reason why Nursing assistance should be started to be individualized according to each need^6^^,^^9^^,^^19^. This shows that a standardized intervention is more effective than conventional care.

The intervention was focused on the subconcepts of structure sources and on the concept of cognitive capabilities, in order to improve the family members' cognitive capacity with the aid of Nursing professionals as credible authorities and with continuing education. The aforementioned method was also used in part by Taboada and Zamora et al^6^^,)(^^7^, obtaining similarly favorable results.

The uncertainty level is always high when the first experiencing the event, as shown in this study^6^. Taking into account the existence of the Hawthorne effect, the study was conducted following the Solomon group methodology, again asserting that the results obtained were not due to the agitation inherent in the family members for knowing what was being studied. This is because the results did not present any significant difference between the experimental groups, nor between the control groups.

Finally, it is important to note the major contribution offered by this study to the phenomenon of uncertainty in family members of patients hospitalized in Intensive Care Units for adults, so that standardized care continues to be replicated and acquires increasingly larger scales and duration in order to better enforce the fundamental right of families to be cared for as set forth in the Colombian political constitution^20^.

As a limitation, the participants present low schooling levels and belong to Stratus 2 or lower, reason why the instrument tended to be somewhat confusing for some people. In addition, the study was developed during the SARS-CoV-2 pandemic; therefore, it necessary to resort to technological aids not mastered by all people, besides having to comply with the mandatory social distancing.

## Conclusions

It is concluded that the intervention is effective in reducing uncertainty among family members of patients hospitalized in an Intensive Care Unit from the city of Neiva, when compared to conventional care: p=0.001. In addition, as it was the first time that the family members had their loved one in an ICU, they presented very high uncertainty levels in all the groups subjected to the baseline assessment, which further reinforces the proposals set forth in Merle Mishel's middle-range theory. This is due to cognitive capacity and to the structure sources, where they initially lacked any credible authority and social support, in addition to their low knowledge about the ICU and their relative's health status.

It should also be considered that Solomon groups were used to avoid the Hawthorne effect, which indicated that the second assessment can be altered by the first one due to prior knowledge about the form. However, such effect was discarded, which indicates that the reduction in uncertainty is due to the intervention.

It is important to conclude that the family members presented higher uncertainty levels due to their limited knowledge about the event; therefore, their level will be high because it is their first time, reason why it would not be necessary to conduct baseline assessments or to use the Solomon group methodology in view of the results obtained.

New research studies in this area should continue to be carried out in the future, in order to keep strengthening the care provided to patients and family members in Intensive Care Units, hospitals and homes.
